# Evaluating a multicomponent program to improve hypertension control in Guatemala: study protocol for an effectiveness-implementation cluster randomized trial

**DOI:** 10.1186/s13063-020-04345-8

**Published:** 2020-06-09

**Authors:** Alejandra Paniagua-Avila, Meredith P. Fort, Russell E. Glasgow, Pablo Gulayin, Diego Hernández-Galdamez, Kristyne Mansilla, Eduardo Palacios, Ana Lucia Peralta, Dina Roche, Adolfo Rubinstein, Jiang He, Manuel Ramirez-Zea, Vilma Irazola

**Affiliations:** 1 Mailman School of Public Health, Columbia University, New York, NY USA; 2 INCAP Research Center for the Prevention of Chronic Diseases, Institute of Nutrition of Central America and Panama – INCAP, Calzada Roosevelt 6-25 zona 11, INCAP III, Guatemala City, Guatemala; 3 Colorado School of Public Health, Aurora, CO USA; 4 Department of Family Medicine, and Adult and Child Center for Health Outcomes Research and Delivery Science, University of Colorado, Aurora, USA; 5 Department of Research in Chronic Diseases, Institute for Clinical Effectiveness and Health Policy (IECS), Buenos Aires, Argentina; 6 Programa Nacional de Enfermedades Crónicas, Ministerio de Salud y Asistencia Social (MSPAS), Guatemala City, Guatemala; 7 Tulane University School of Public Health and Tropical Medicine and Tulane University Translational Science Institute, New Orleans, LA USA

**Keywords:** Multicomponent program, Hypertension, Cardiovascular disease, Primary care, Health systems, Implementation strategies, Implementation science, Low-income and middle-income countries, Guatemala, Non-communicable diseases

## Abstract

**Background:**

Hypertension is a major risk factor for cardiovascular disease (CVD). Despite advances in hypertension prevention and treatment, the proportion of patients who are aware, treated and controlled is low, particularly in low-income and middle-income countries (LMICs). We will evaluate an adapted version of a multilevel and multicomponent hypertension control program in Guatemala, previously proven effective and feasible in Argentina. The program components are: protocol-based hypertension treatment using a standardized algorithm; team-based collaborative care; health provider education; health coaching sessions; home blood pressure monitoring; blood pressure audit; and feedback.

**Methods:**

Using a hybrid type 2 effectiveness-implementation design, we will evaluate clinical and implementation outcomes of the multicomponent program in Guatemala over an 18-month period. Through a cluster randomized trial, we will randomly assign 18 health districts to the intervention arm and 18 to enhanced usual care across five departments, enrolling 44 participants per health district and 1584 participants in total. The clinical outcomes are (1) the difference in the proportion of patients with controlled hypertension (< 130/80 mmHg) between the intervention and control groups at 18 months and (2) the net change in systolic and diastolic blood pressure from baseline to 18 months. The context-enhanced Reach, Efficacy, Adoption, Implementation, Maintenance (RE-AIM)/Practical Robust Implementation and Sustainability Model (PRISM) framework will guide the evaluation of the implementation at the level of the patient, provider, and health system. Using a mixed-methods approach, we will evaluate the following implementation outcomes: acceptability, adoption, feasibility, fidelity, adaptation, reach, sustainability, and cost-effectiveness.

**Discussion:**

We will disseminate the study findings, and promote scale up and scale out of the program, if proven effective. This study will generate urgently needed data on effective, adoptable, and sustainable interventions and implementation strategies to improve hypertension control in Guatemala and other LMICs.

**Trial registration:**

ClinicalTrials.gov: NCT03504124. Registered on 20 April 2018.

## Background

Hypertension is the leading preventable risk factor for cardiovascular disease (CVD), premature death and disability worldwide [1]. It contributes to the burden of cardiovascular disease and chronic kidney disease worldwide, particularly in low-income and middle-income countries (LMICs) [2, 3]. It is estimated that 31.1% of the adult population had hypertension in 2010, three quarters of whom were living in LMICs [3]. While its prevalence is steady or decreasing in high-income countries, it increased by 7.7% from 2000 to 2010 in LMICs [3, 4]. In Latin America, hypertension is the most important risk factor for coronary heart disease and stroke [5]. However, the proportion of patients who are aware, treated and controlled is low. A survey conducted in Guatemala showed that the prevalence of hypertension in adults older than 40 years is 41%, while only 61% are aware of their condition and only half of those who are aware usually take antihypertensive medications [6].

Despite the availability of evidence-based hypertension treatment guidelines, multiple barriers hinder the appropriate management of hypertension in primary care settings. Hypertension guidelines recommend antihypertensive medications and individualized lifestyle changes, which include weight loss, physical activity, reduced alcohol and sodium intake, and a diet rich in fruits and vegetables and in low-fat dairy products with reduced saturated and total fat (Dietary Approaches to Stop Hypertension, DASH) [7–12]. Our formative needs assessment documented several limitations related to hypertension management in Guatemala, including a limited health budget for the treatment of non-communicable diseases, fragmented governance and service delivery, inadequate training of the healthcare workforce, and shortage of essential hypertensive medications and basic equipment, particularly at frontline facilities [13]. In addition, an overarching challenge is the prioritization of infectious diseases and maternal and child health over non-communicable diseases [13]. In other countries, barriers for implementing hypertension treatment guidelines at the primary care level include organizational-level obstacles, communication problems between the primary and secondary levels of care, multiple competing demands on physicians’ time, and lack of reimbursement for preventive counseling [14, 15]. Many implementation strategies targeting healthcare administration, facilities, providers, and patients have been proven effective at improving hypertension control. Specifically, these strategies include team-based care, health coaching sessions, home-based blood pressure (BP) monitoring, clinical decision support, BP audit and feedback, and training of healthcare providers. Moreover, a combination of strategies is more effective than individual ones [16].

This study is an implementation-effectiveness, hybrid, type 2, cluster randomized control trial that will evaluate a multilevel and multicomponent hypertension control program within the Guatemalan primary care system [17]. Through a formative mixed-methods assessment and adaptation workshops, we have adapted the effective Hypertensive Control Program in Argentina (HCPIA) and other implementation strategies to the Guatemalan context [13]. The multicomponent program includes a protocol-based hypertension treatment and five implementation strategies: team-based collaborative care, health provider education, health coaching sessions, home blood pressure monitoring, and blood pressure audit and feedback. This program targets the first level (health posts) and second level (health centers) of care in the public health system.

## Methods and design

### Setting

The Guatemalan public health system serves 70% of the population and is organized in three levels of care [18]. The first, second, and third levels comprise health posts, health centers, and hospitals, which serve the community, municipal, and the regional level, respectively. Health posts are staffed by auxiliary nurses, while health centers are staffed by general physicians, professional nurses, auxiliary nurses and, in some cases, psychologists or social workers. Health posts and health centers are responsible for providing promotional, preventative, and primary care services. Health districts represent the municipal administration. The three levels of care are connected by referral networks with the goal of decentralizing health services and increasing access to care. However, the vast majority of healthcare providers and facilities are concentrated in urban areas, leaving rural communities with limited access to health services [18].

**Fig. 1:**
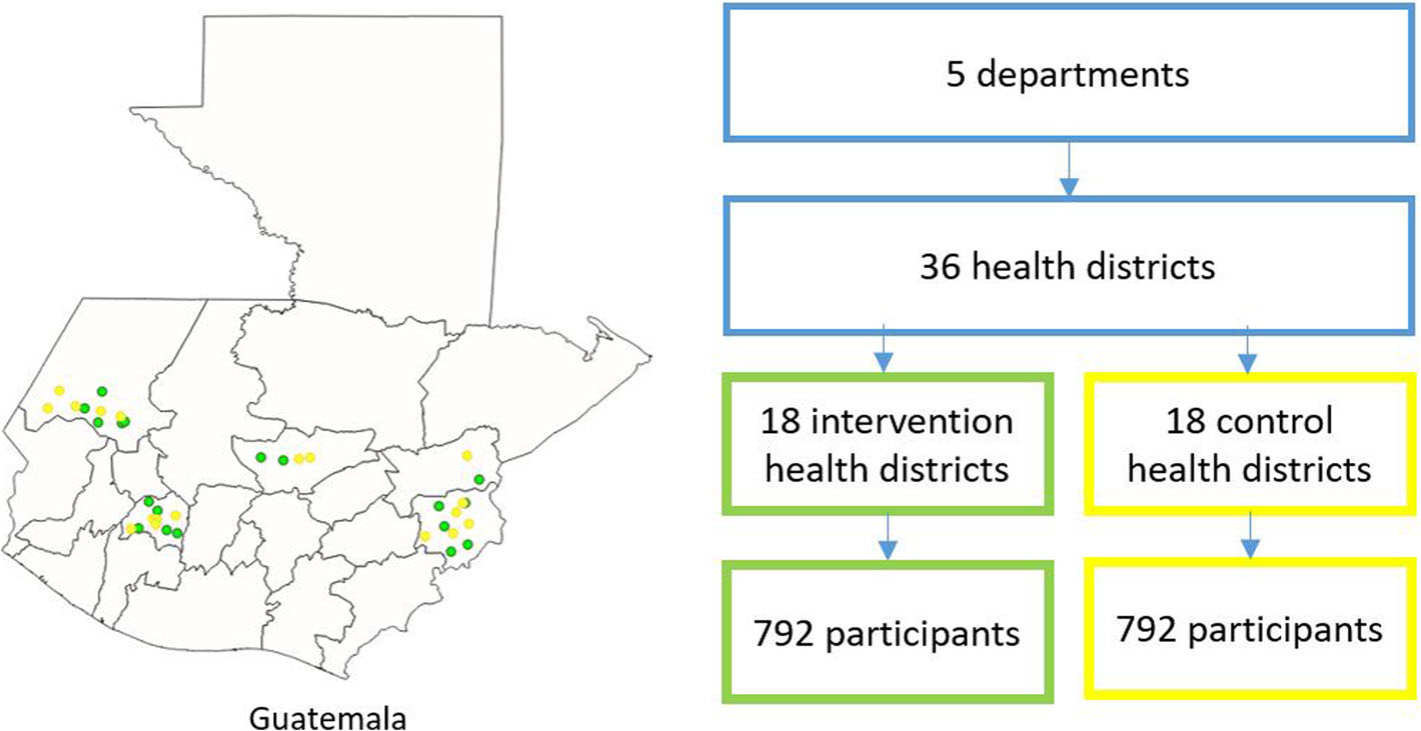
Study sites: intervention and control health districts

We are conducting this study within the first (health posts) and second (health centers) levels of care. With approval from the Ministry of Health, we selected 36 health districts distributed in five departments: Baja Verapaz (*n* = 4), Chiquimula (*n* = 10), Huehuetenango (n = 10), Sololá (n = 10), and Zacapa (*n* = 2). The study will be implemented at the health center and 1–2 health posts per health district, making a total of 36 health centers and 71 health posts (See Fig. 1).

The eligibility criteria for health districts are the following:

1. Having at least one health post with two or more auxiliary nurses and basic infrastructure to store clinical charts
2. Serving rural and semirural communities
3. Having at least one professional nurse or physician per health district, responsible for supervising the health post(s)

### Aims

The overarching aim of this study is to evaluate the clinical effectiveness and implementation outcomes of a hypertension control multicomponent program within the first and second levels of care in Guatemala, compared to usual care. Our main hypothesis is that the multicomponent program will improve hypertension control among patients with uncontrolled hypertension treated in the public healthcare system of Guatemala.

The co-primary objectives are:

1. To test if a multilevel and multicomponent intervention program improves hypertension control among Guatemalan hypertensive patients over an 18-month period compared to usual care.
2. To evaluate the acceptability, adoption, feasibility, fidelity, adaptation, reach, and sustainability of implementing the intervention in the primary care setting.

The secondary objective is

3. to evaluate the cost-effectiveness of the multilevel and multicomponent intervention program, compared to usual care.

### Study design: implementation-effectiveness cluster randomized controlled trial

**Fig. 2:**
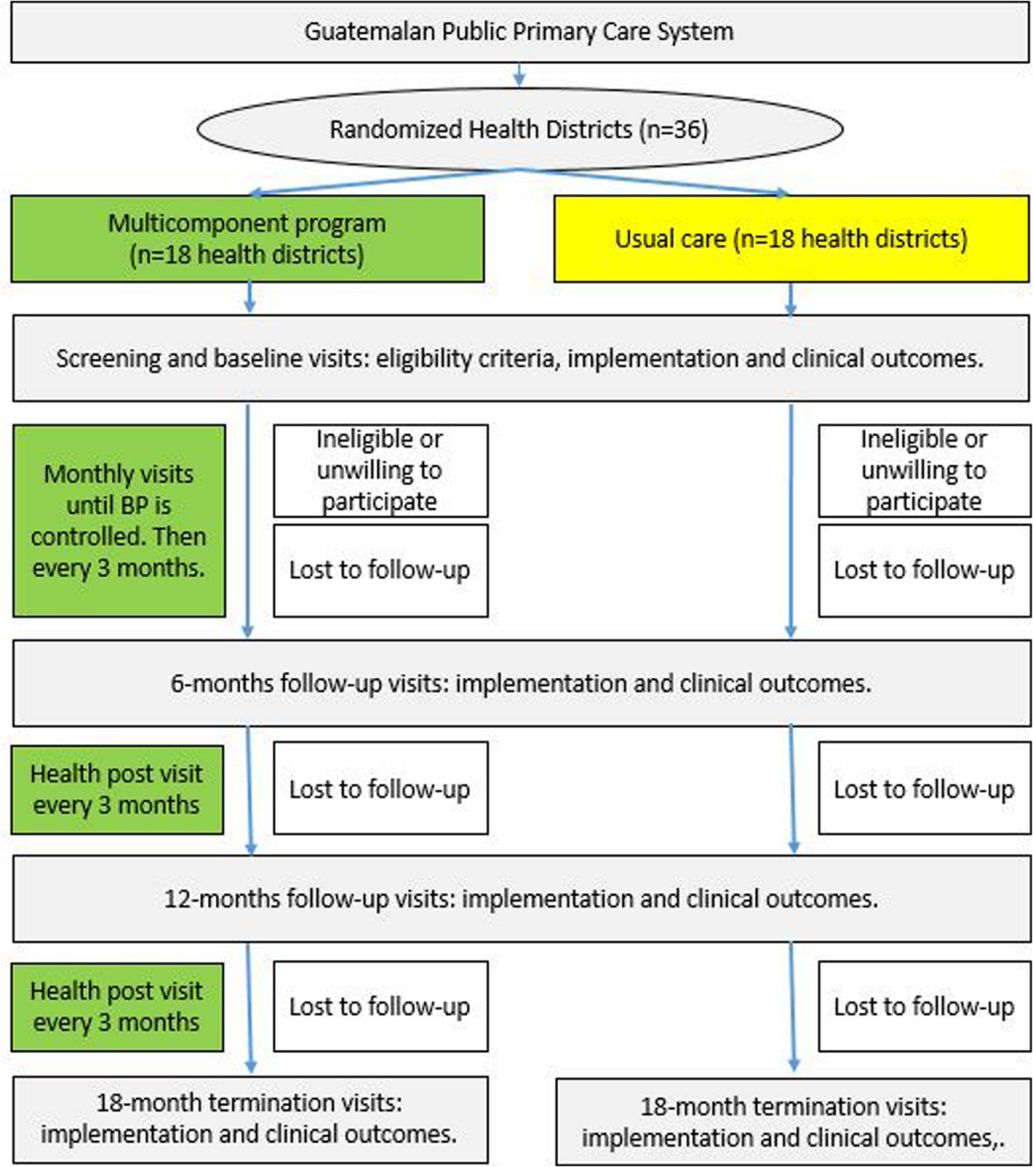
Trial flow chart. BP, blood pressure

**Fig. 3:**
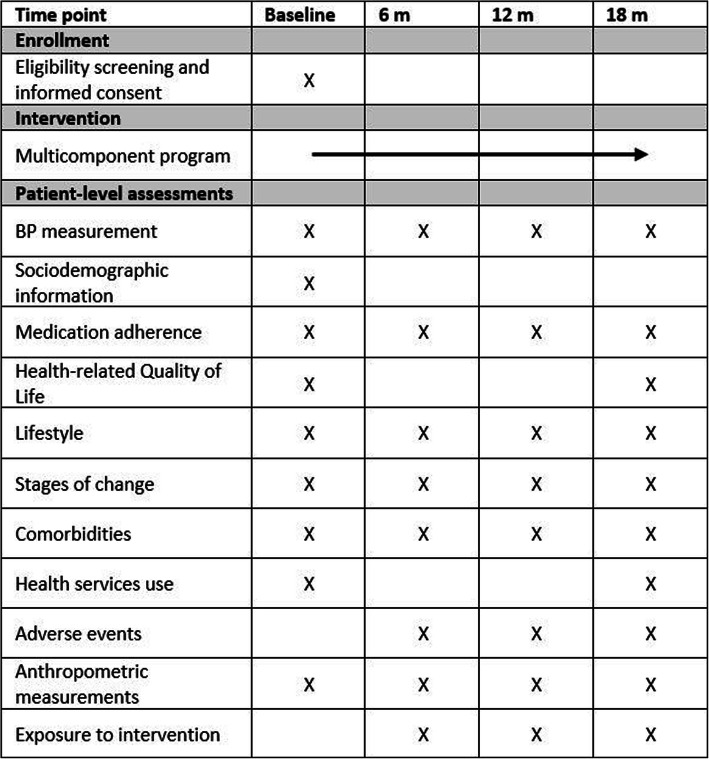
Standard Protocol Items: Recommendation for Interventional Trials (SPIRIT) figure

We are conducting a hybrid type 2 effectiveness-implementation, cluster randomized controlled trial (cRCT). We have randomly assigned 18 health districts (clusters) to the intervention arm and 18 to enhanced usual care (control arm) across five departments. We will enroll 44 participants per health district and 1584 participants in total. After selecting 36 eligible health districts, and before initiating participant recruitment, health districts were randomized and stratified by department, using a computerized random number generator. The trial flow chart is shown in Fig. 2 and the Standard Protocol Items: Recommendation for Interventional Trials (SPIRIT) figure is shown in Fig. 3. The SPIRIT checklist is provided in Additional file 1.

### Study participants

The study follows minimum eligibility criteria to evaluate the intervention in a real-world setting**.** Men and women 40 years or older with uncontrolled hypertension, and who meet the following eligibility criteria will participate in the study:

- Have uncontrolled hypertension, which will be ascertained by measuring BP at two screening visits, scheduled 1–7 days apart from each other. Participants with stage II hypertension (average systolic BP ≥ 140 mmHg or diastolic BP ≥ 90 mmHg) are eligible. Participants with stage I hypertension (average systolic BP 130–139 mmHg or diastolic BP 80–89 mmHg) are eligible if they meet at least one of the following characteristics:
  1. taking antihypertensive medications;
  2. history of cardiovascular disease (myocardial infarction or stroke);
  3. estimated cardiovascular risk higher than 10% in 5 years (based on the NHANES I follow-up study cardiovascular risk estimation) using a non-invasive prediction indicator [19, 20].
- Live in a community served by one of the 71 participating health posts and willing to receive hypertension care at the health post.
- Be willing to sign an informed consent form before any study procedure is performed. For illiterate patients, a witness who reads and understands the consent will co-sign the informed consent form.

Individuals who have any of the following exclusion criteria will not be eligible to participate in the study:

- Pregnant according to self-report
- Diagnosed end-stage renal disease or any chronic terminal disease
- Bedridden
- Planning to move from the study area within the next 18 months

Participants are being recruited from participating health posts and from the community in their catchment area. Auxiliary nurses help study staff to identify potential participants and implement the intervention (see below), but do not participate in any study measurement.

### Multicomponent intervention

**Fig. 4:**
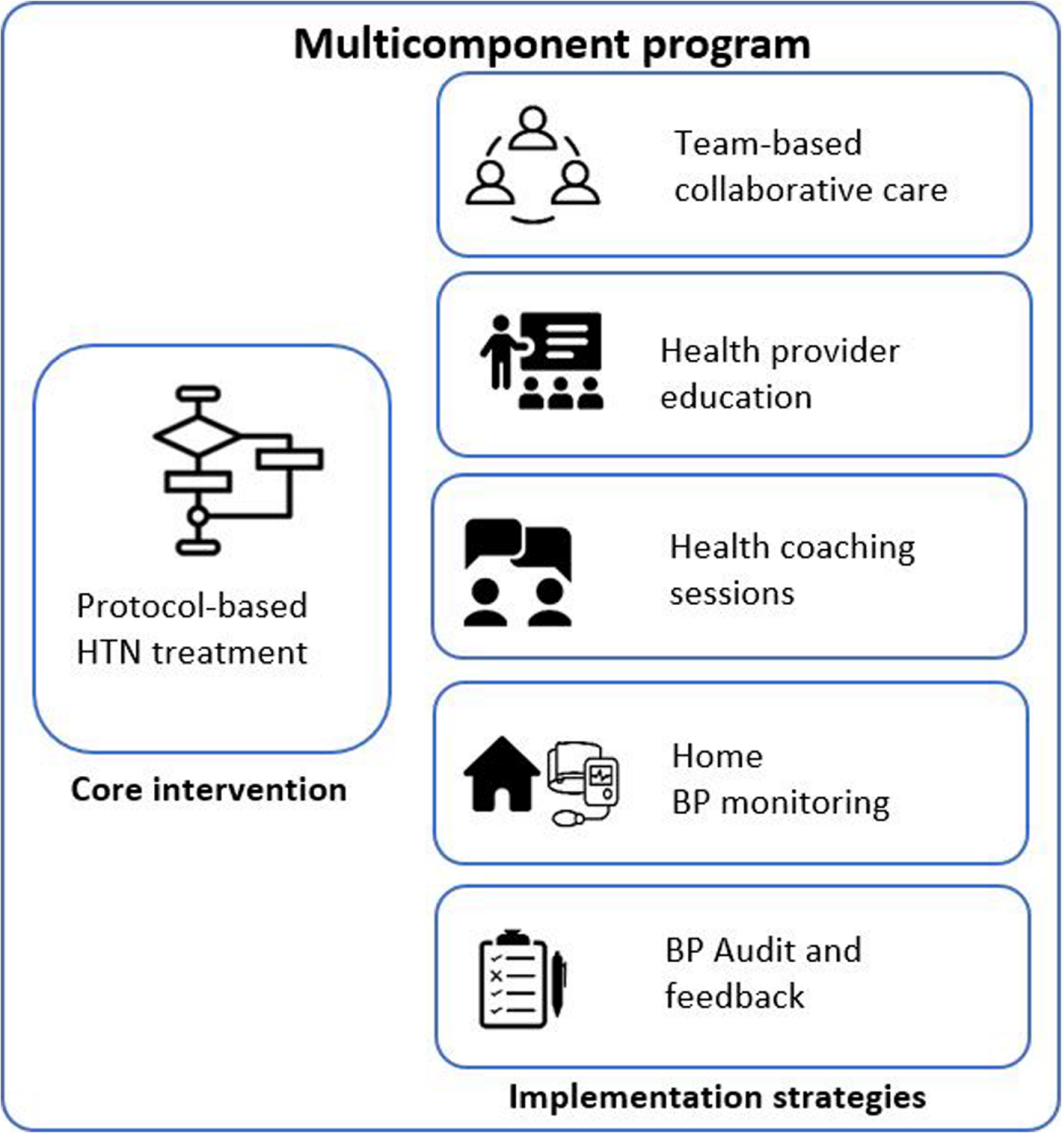
Multicomponent hypertension control program: core intervention and implementation strategies. HTN, hypertension; BP, blood pressure

The study intervention is a multicomponent and multilevel program to improve hypertension control over 18 months. The program is composed of one core intervention and five evidence-based implementation strategies (See Fig. 4), which are defined as methods to enhance the adoption, implementation, and sustainment of the core intervention [21]. The core intervention and implementation strategies were previously adapted to the rural Guatemalan context by the study team and stakeholders from the Ministry of Health and local communities [13]. Physicians and nurses working at intervention health centers and auxiliary nurses working at health posts are responsible for delivering the intervention.

### Core intervention: protocol-based hypertension treatment

The study team and Ministry of Health officials designed a standardized stepped-care hypertension treatment protocol summarized in an algorithm, based on the American Heart Association (AHA) Hypertension Guidelines 2017 and the Guatemala Ministry of Health Healthcare Norms 2018 [22, 23]. After participants are enrolled in the study, health district physicians, nurses, and auxiliary nurses will establish an individualized treatment plan for the participant to reach a BP target < 130/80 mmHg, with a combination of anti-hypertensive medications offered by the Ministry of Health: hydrochlorothiazide, enalapril and losartan. The study team provided educational materials and pocket cards summarizing the hypertension treatment algorithm to healthcare providers and an electronic BP monitor (Omron HEM-7121) to each health center and health post.

### Implementation strategies

#### Team-based collaborative care

Teams of physicians, nurses, and auxiliary nurses from health posts and health centers will work collaboratively to establish a treatment plan for hypertensive patients. After study enrollment, a physician or nurse will perform a physical examination, confirm the hypertension diagnosis and select the initial anti-hypertensive medications following the standardized hypertension treatment protocol described above. Auxiliary nurses from health posts (first level of care) will be in charge of follow up and health coaching sessions, and will coordinate and connect patients with physicians and nurses at the health center (second level of care). The collaborative team will meet at least monthly at the health center to discuss cases of uncontrolled hypertension or adverse events and make clinical decisions following the standardized hypertension treatment protocol. Usual care provided by the Ministry of Health for patients with hypertension does not include team-based collaborative care.

#### Health provider education

The study team provided an interactive 2-day workshop for physicians, nurses and auxiliary nurses, during the second semester of 2019. Training content included: BP management using a stepped-care protocol-based hypertension treatment; titration and adverse effects of anti-hypertensive medications; team-based collaborative care; and motivational interviewing skills to promote medication adherence and healthy lifestyle modifications during health coaching sessions. One month after the training, the study team conducted individualized field certifications on blood pressure measurement and health coaching sessions with auxiliary nurses working in health posts. Periodic training will be provided to newly hired providers and as refreshers. Usual hypertensive care does not include training for healthcare providers about hypertensive management.

#### Health coaching sessions

Auxiliary nurses conduct health coaching sessions focused on promoting adherence to anti-hypertensive medications, strategies to overcome treatment side effects and lifestyle modifications: reaching or maintaining a healthy weight, limiting sodium and alcohol intake, getting regular physical activity, and adopting an eating plan based on the Dietary Approaches to Stop Hypertension (DASH). Participants receive an educational flipchart adapted from the manual “Healthy and Happy Heart” (*Corazon Sano y Feliz),* previously developed and piloted in Guatemala [24], and a card to register BP measurements. Relatives will be encouraged to participate in health coaching sessions. During the first 3 months of the intervention, health coaching sessions will take place monthly during the first 3 months of the intervention. If the patient meets the BP target, the frequency will be reduced to every 3 months. Usual hypertensive care does not include health coaching sessions.

#### Home blood pressure monitoring

After study enrollment, each patient receiving care at one of the intervention health districts obtains an electronic home BP monitor that stores 30 readings with date and time stamp (Omron HEM-7121). Auxiliary nurses will teach patients and literate relatives to measure BP using the electronic monitor and document readings on a card provided by the study team. Auxiliary nurses will review the patient card and document mean home BP-readings during the health coaching sessions, which the care team will use to guide hypertension management decisions. Home BP monitoring is not part of usual hypertensive care.

#### Blood pressure audit and feedback

Auxiliary nurses create lists of hypertensive patients documenting their anti-hypertensive medications, adverse events, and their controlled or uncontrolled status. Then, auxiliary nurses take these lists to collaborative team meetings, where the group reviews cases and makes management decisions following the standardized hypertension treatment protocol. Given that usual hypertensive care does not include completion of patient charts, the study team is providing paper-based forms for auxiliary nurses to generate the lists of patients with hypertension. Blood pressure audit and feedback is not included in usual hypertensive care.

### Enhanced usual hypertensive care

Healthcare providers based at the control health districts will receive a one-morning, 4-h training session on the Ministry of Health Healthcare Norms 2018 for hypertension management, conducted by Ministry of Health representatives. Similar to the intervention arm, the study will provide one electronic BP monitor (Omron HEM-7121) to each health center and health post. At the central government and department levels, the study team will work with Ministry of Health officials to promote the purchase, distribution and availability of essential hypertensive medications at participating health districts at a minimum. While participants in the intervention group receive an electronic BP monitor (Omron HEM-7121) at the first study visit, those in the control arm will receive the BP monitor and study-specific educational materials at the last study visit.

### Outcomes

The primary clinical outcome is the 18-month difference in the proportion of participants with controlled hypertension (BP < 130/80 mmHg) between the intervention and control groups. The secondary clinical outcome is the 18-month net change in systolic and diastolic BP from baseline. The BP measurement for inclusion in the study and used in the outcome analysis will be standardized following the AHA guidelines and conducted by trained study staff [22, 25]. The clinical outcomes correspond to intervention effectiveness, measured at the individual participant level. We will also measure implementation outcomes as part of the second co-primary aim. The context-enhanced Reach, Efficacy, Adoption, Implementation, Maintenance (RE-AIM)/ Practical Robust Implementation and Sustainability Model (PRISM) framework will guide the evaluation of the implementation at the patient, provider, and health system levels. Using a mixed-methods approach, we will evaluate the following implementation outcomes: acceptability, adoption, feasibility, fidelity, adaptation, reach, sustainability, and cost effectiveness [26].

### Sample size and power

The power calculation for the primary outcome was based on the following assumptions: (1) a two-sided significance level of 0.05; (2) statistical power of 90%; (3) a proportion of patients with BP < 130/80 mmHg of 50% in the control group; (4) detectable group differences in proportion of BP < 130/80 mmHg of 15% (65% of patients with BP < 130/80 mmHg in the intervention group); (5) intra-cluster correlation (ICC) coefficient for hypertension control of 0.055; (6) 18 clusters (health districts) per group; and (7) 85% follow-up rate by 18 months. The sample size for each cluster is 37 based on a two-sample *Z* test for individual-level comparison of a cluster design. Further assuming an 85% follow-up rate by 18 months, we will need to recruit 44 participants from each district and 1584 study participants for the entire study. The statistical power is even higher for the secondary outcomes because they are continuous variables. Table 1 shows the statistical power based on various follow-up rates and ICCs. The intra-cluster correlation over 18 months was based on our data from the Hypertensive Control Program in Argentina (HCPIA) [27, 28].

**Table 1:** Power calculation

Follow-up rate	Sample size for analysis (total/per health district)	Intra-class correlation					
		0.050	0.055	0.060	0.065	0.070	0.075
80%	1260/35	0.9132	0.8961	0.8791	0.8614	0.8434	0.8524
81%	1296/36	0.9154	0.8987	0.8821	0.8631	0.8453	0.8275
83%	1332/37	0.9176	0.9013	0.8836	0.8665	0.8490	0.8295
85%	1368/38	0.9197	0.9038	0.8865	0.8681	0.8508	0.8336

a = 0.05, control rate in control group = 50%, difference in control rate between intervention and control groups = 15%, total sample size = 1584 participants

We expect that each health district will enroll at least 44 participants. Given the longstanding engagement of Ministry of Health providers at the community level, we anticipate being able to successfully enroll the total number of participants. To enhance recruitment of participants, we have engaged healthcare providers and community leaders since the preparation phase of the trial. In addition, healthcare providers were familiarized with eligibility criteria and the enrollment process during training workshops and are referring potential study participants to study staff, who maintain constant communication with providers.

### Statistical analysis plan

The primary analysis will be conducted on an intention-to-treat basis. We will compare the proportion of participants who achieve BP control in the intervention arm and the control arm by using logistic mixed-effects regression analysis, where participants and clusters are included as random effects and the intervention group, time, and group-by-time interaction are included as fixed effects. In a secondary analysis, blood pressure values at baseline, 6 months, 12 months, and 18 months will be modeled in a linear mixed-effects regression analysis. Pre-defined subgroup analyses by age (< 60 vs. ≥ 60 years), sex (men vs. women), history of cardiovascular disease (CVD) (yes vs. no), and body mass index (< 30 vs. ≥ 30 kg/m^2^) will be conducted. Further details of the data management, statistical methods, and quality control plans are available upon request from the authors.

### Implementation evaluation

We will use the context-enhanced RE-AIM/PRISM framework to evaluate the implementation of the multicomponent program [29, 30]. The implementation evaluation will allow us to monitor and improve program implementation, understand the relationship between implementation characteristics and health outcomes, and design the dissemination plan if the program is proven effective. We will assess the expanded RE-AIM/PRISM dimensions at the patient, provider, and health system levels (See Table 2).

**Table 2:** Implementation evaluation

RE-AIM/ PRISM dimension	Patient level	Healthcare provider (HCP) level	System level
**Reach**	Number of participants per health district Representativeness of target population		
**Effectiveness**	See Fig. 3		
**Adoption**		Number of HCPs who participate in training sessions/total HCPs Representativeness of HCPs who participate in training sessions	Number of health districts, health centers and posts that participate in training sessions/total Representativeness of health districts that participate in training sessions
**Implementation (fidelity, adaptation)**	Documentation of home-based BP measurement on patient’s card Number of health coaching sessions received	Characteristics of training workshops for HCPs Application of HTN algorithm by HCP Extent to which implementation strategies (patient lists, audit and feedback, collaborative team meetings, supervision, and coaching sessions) are implemented Specific modes of implementation by different providers Types and frequency of delivery adaptation made	Availability of intervention inputs: medications, monitors, materials, staff Specific modes of implementation in different sites
**Maintenance (sustainability)**	Sustained BP control, medication adherence, lifestyle changes, quality of life, stages of behavior change at 18 months	Sustained HTN knowledge over time Intention to continue implementation beyond study period Adaptations needed in order to continue implementation	Intention to continue beyond study period Adaptations needed in order to continue implementation
**Fit**		Adaptations by healthcare providers to make the intervention and implementation strategies fit to their context	Changes at the national, state and health district level to be able to implement the intervention and implementation strategies
**Sustainability infrastructure**		Policies or programs related to HCP hiring, training or retention Supervision infrastructure Audit and feedback	Support and resources from MOH leadership to implement intervention Medication availability Information system BP monitors Staffing

*HTN* hypertension, *BP* blood pressure, *MOH* Ministry of Health

In addition to the five dimensions of RE-AIM (reach, effectiveness, adoption, implementation and maintenance) we will assess the program fit and sustainability infrastructure of PRISM [31]. The implementation outcomes that we will measure are: acceptability, adoption, feasibility, fidelity, adaptation, reach, sustainability, and cost effectiveness [26].

We will use a combination of quantitative and qualitative methods to assess the domains of interest. We will gather data during patients’ study visits at 6, 12, and 18 months. In addition, we will make regular (1–2 months) visits to healthcare facilities to capture study inputs and ongoing program implementation captured in checklists. In a subset of study sites, we will conduct interviews with participants and family members, providers, and public health administrators using semi-structured interview guides combined with chart-stimulated recall, shadowing, and direct observation.

### Cost-effectiveness analysis

We will perform a cost-effectiveness analysis using the individual patient data collected at follow-up visits (see Fig. 3), expressed as incremental cost per additional percentage of patients that achieved hypertension control at 18 months. Intervention costs will include fixed costs such as education of health providers and salary of auxiliary nurses, and variable costs such as electronic BP monitors. Healthcare costs will include ambulatory costs, such as drugs and laboratory tests, and hospital care (hospitalization). Protocol-driven costs will be excluded. We will analyze differences in costs following a similar analytical approach as that used for estimating health outcomes [32]. Uncertainty around the incremental cost-effectiveness ratio (ICER) will be estimated by bootstrapping techniques, and a 95% credible interval will be reported [33, 34].

## Discussion

This is the first randomized cluster trial in Central America to test the effect of a multicomponent intervention program for BP control in underserved rural populations. The intervention and study outcomes are patient-centered, and patients, Ministry of Health provider-teams, and other stakeholders have been engaged at every step of the proposed study. The multicomponent intervention program is designed to address barriers at the healthcare system, provider-team, and patient levels. The proposed study will generate urgently needed data on effective, adoptable, and sustainable intervention strategies aimed at reducing BP-related disease burden in Central America and other low-income settings.

Although the efficacy and effectiveness of lifestyle modifications and antihypertensive drug treatment on the prevention of HTN and consequent CVD risk have been demonstrated in randomized controlled trials, this knowledge has not been fully applied in LMIC [35, 36]. The proposed study will test whether an evidence-based, multilevel and multicomponent intervention program can be translated to and is feasible in the primary healthcare systems of this region.

We will disseminate the study findings, promote scale up, and scale out of the program, if proven effective. This study will generate urgently needed data on effective, adoptable, and sustainable intervention and implementation strategies to improve hypertension control in Guatemala and other low- and middle-income countries.

## Trial status

A stakeholder engagement process and needs assessment are finalized. The study manual of operations was developed and training of study staff has been completed. The Data Safety and Monitoring Board met twice during 2019: prior to study enrollment and during the first semester of enrollment. Intervention educational materials for healthcare providers and patients were adapted and finalized. Training workshops and field certifications of healthcare providers were developed and completed. The Community Advisory Board was formed with local healthcare providers and hypertensive patients and has met twice. Enrollment into the study began in July 2019 and 89% was completed by March 20th 2020. Enrollment has been paused due to COVID-19 and will reinitiate as soon as national policies allow. This is study protocol version 6.1 and the version date is 21 May 2019.

## Supplementary information

**Additional file 1.** SPIRIT 2013 Checklist: Recommended items to address in a clinical trial protocol.

## Data Availability

Upon completion of the trial, datasets used and analyzed during the study are available from the corresponding author on reasonable request.
